# SARS-CoV-2 Possible Etiology of Cerebral Venous Thrombosis in a Teenager: Case Report and Review of Literature

**DOI:** 10.3390/v15020405

**Published:** 2023-01-31

**Authors:** Ioana Grigore, Ingrith Miron, Cristina Gavrilovici, Vasile Valeriu Lupu, Dorin Cristian Antal, Thomas Gabriel Schreiner, Catalin Prazaru, Ancuta Lupu, Felicia Dragan, Ecaterina Grigore

**Affiliations:** 1 “St. Mary” Children Emergency Hospital, 700309 Iasi, Romania; 2 Pediatrics, “Grigore T. Popa” University of Medicine and Pharmacy, 700115 Iasi, Romania; 3 Neurology, “Grigore T. Popa” University of Medicine and Pharmacy, 700115 Iasi, Romania; 4 Faculty of Medicine and Pharmacy, University of Oradea, 410087 Oradea, Romania; 5 Faculty of General Medicine, “Grigore T. Popa” University of Medicine and Pharmacy, 700115 Iasi, Romania

**Keywords:** SARS-CoV-2 infection, teenager, cerebral thrombosis, child

## Abstract

Cerebral venous thrombosis in pediatric patient has a varied etiology. The authors present the case of a teenager who, since the debut of SARS-CoV-2 infection, has accused intermittent right side hemicrania, which has become persistent in association with nausea and vomiting since the 5th day of quarantine. She was hospitalized in the 9th day since the debut. Neuroimaging revealed extended venous cerebral thrombosis affecting the right sigmoid sinus, the transverse sinus bilaterally, the confluence of the transverse sinuses and the right internal jugular vein. The evolution was favorable under anticoagulant and symptomatic treatment. Laboratory tests excluded other etiological causes for the cerebral venous thrombosis, thus the authors consider that cerebral thrombosis is a possible complication of SARS-CoV-2 infection in teenagers.

## 1. Introduction

Cerebral venous thrombosis is a rare condition in pediatric patients, with an incidence of 0.07–0.14/10,000 children [1,2]. Studies report a higher frequency in neonates (30–50%) compared to other age groups. Moreover, approximately 2/3 of the cases are reported in males [3]. At the present time, the recognition of cerebral venous thrombosis has risen together with the use of neuroimaging techniques in pediatric patients.

The etiology of cerebral venous thrombosis in children is multifactorial and clinical findings are not specific. In these conditions, neuroimaging techniques are essential for an accurate diagnosis in the cases where there is significant clinical suspicion of cerebral venous thrombosis. Moreover, investigations of the prothrombotic status such as testing for factor V Leiden mutations, deficits in protein C, protein S, and antithrombin levels are of great importance.

Besides supportive care (hydro-electrolyte balance) and symptomatic treatment (antibiotics in case of infection, antiepileptic treatment in case of seizures, correction of anemia), the treatment consists of intravenous unfractionated heparin or subcutaneous low molecular weight heparin, followed by the administration of oral antithrombotic drugs for 3 to 6 months [4]. Management of cerebral venous thrombosis in children is controversial because there is no general agreement regarding the antithrombotic treatment. Some authors recommend intravenous unfractionated heparin or subcutaneous low molecular weight heparin, other authors recommend the use of antiplatelet agents (acetylsalicylic acid or dipyridamole) in cerebral venous thrombosis in children [5,6].

Numerous cases of atypical pneumonia with unknown etiology were reported beginning with December 2019 in Wuhan region, China, and the new coronavirus (SARS-CoV-2) was identified in January 2020. It is this virus that is responsible for a severe acute respiratory syndrome within the condition named COVID-19. The most common symptoms of this disease in children are related to the respiratory system (including coughing, rhinorrhea, dysphagia, pharyngitis), fever and gastrointestinal manifestation (vomiting, diarrhea, abdominal pain and difficulty in feeding in small children) [7]. Some authors are of the opinion that SARS-CoV-2 infection is less severe in children and that neurological complications caused by the virus are more frequent in adults [7]. In general, neurological complications reported in patients of pediatric age are less frequent and include headaches and loss of smell and/or taste [8]. In a study which reviews literature data and focuses on both the frequency and the characteristics of neurological complications in children aged under 18 years old, Panda et al. (2020) discovered nonspecific neurological symptoms (headache, myalgia and fatigue) in around 16% of children and specific neurological complication represented by seizures and encephalopathy in approximately 1% cases. The authors concluded that neurological complications are generally rare in children with COVID-19 [9]. However, there are studies in which it is stated that neurological manifestation of SARS-CoV-2 in children can be severe, for instance encephalitis, seizures, acute cerebrovascular disease, transverse myelitis, Guillain-Barre syndrome, benign intracranial hypertension, meningoencephalitis and acute disseminated encephalomyelitis [10,11,12].

The authors present the case of an adolescent who was diagnosed with cerebral venous thrombosis secondary to SARS-CoV-2 infection. The aim of this case study is to draw attention to the fact that there is a possible association between these two conditions in pediatric patients, even though it has not been widely reported in the reviewed medical literature. This association has been frequently reported in adults [13].

## 2. Case Report

A female teenager (17 years and 2 months of age) tests positive for the molecular detection of SARS-CoV-2 after having seen the general practitioner (GP) for headache (right hemicrania type), rhinorrhea, coughing, and intense fatigue. She is diagnosed with a mild COVID-19 infection and the recommended treatment by GP includes usual analgesic drugs (Paracetamol 500 mg × 3/day), vitamins and dietary supplements (vitamin C 1000 mg/day, vitamin D3 1000 UI/day, zinc 75 mg/day, omega3 1000 mg/day) and quarantine at home. The hemicrania becomes persistent on the 5th day of quarantine, being associated with nausea and vomiting. Moreover, the patient presents diarrheic stools, for which she receives appropriate hygienic-dietary regimen recommended by GP. On the 9th day of quarantine, she comes to the hospital for persistent right hemicrania and is hospitalized in the Department of Pediatric Neurology. It is worth mentioning that the patient was not vaccinated antiCovid19 and denied using either oral contraceptive pills or any other chronic treatment. Family history does not reveal neither neurological nor thrombotic conditions. Moreover, her personal medical history was not of great relevance, apart from irregular and prolonged menstrual bleeding for which she had been evaluated by an endocrinologist, without receiving any specific treatment.

The general clinical exam showed altered general condition, serous rhinorrhea, semi productive cough without modifications at pulmonary auscultation, normal cardiologic exam. She was afebrile, oxygen saturation was 99%, respiratory rate was 20 per minute, blood pressure was 110/70 mmHg and heart rate was 82 beats per minute. By means of anamnesis it was discovered that she had slow intestinal transit (absent stool for 3 days) after she receives appropriate hygienic-dietary regimen for the previous signs of gastroenteritis. The neurologic exam was objectively normal, without any neurological deficits, revealing right hemicrania with an intensity of 9/10, with no phono- or photosensitivity. There were no signs of meningeal irritation either.

At admission the complete blood count revealed leukocytosis with neutrophilia, lymphopenia, monocytosis, and reactive thrombocytosis. Liver function tests and renal function tests were normal, except for a higher value of direct bilirubin, but normal levels of total bilirubin and indirect bilirubin. The seric electrolytes (Na, K, Ca, P) were normal, but there were high values of C-reactive protein and ferritin. Coagulation tests revealed high values of D-Dimer and low values of the activated partial thromboplastin time. All of the abnormal values had reached the normal ranges over time.

The abdominal ultrasound suggested no pathological modifications, apart from diffuse abdominal meteorism, and the thoracic X-ray indicated interstitial pneumonia.

The cardiac ultrasound revealed mildly dilated coronal arteries, minimal aortic and pulmonary regurgitation, and minimal pericardial effusion.

**Figure 1:**
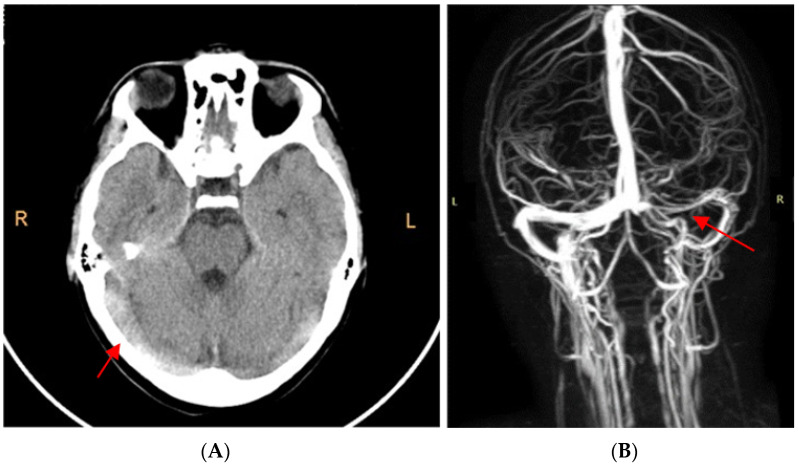
(**A**). CT scan without contrast: highlighted hyperdensity of the internal cerebral veins raised the suspicion of cerebral venous thrombosis. (**B**). MRI angiography: extended cerebral venous thrombosis affecting the right sigmoid sinus, both transverse sinuses, their point of confluence, and the right internal jugular vein.

The cerebral computed tomography (CT) scan highlighted spontaneous hyperdensity of the right sigmoid sinus, both transverse sinuses and their point of confluence (Figure 1A). Agenesis of the right frontal sinus and left frontal and bilateral maxillary sinusitis were also present.

Cerebral magnetic resonance angiography confirmed the extended cerebral venous thrombosis affecting the right sigmoid sinus, both transverse sinuses, their point of confluence, and the right internal jugular vein (Figure 1B).

Extended laboratory tests were run after the diagnosis of cerebral venous thrombosis. Antiphospholipid antibodies, Antinuclear Antibody Test 14 (ANA Test 14), and lupus anticoagulant were negative. Antithrombin III, protein S, and protein C were in normal range (those tests had been carried out before the antithrombotic treatment was initialized). Mutations of methylenetetrahydrofolate reductase genes (MTHFR) and prothrombin genes were absent.

The final diagnosis of our patient was extended cerebral venous thrombosis, headache, interstitial pneumonia, fronto-maxillary sinusitis, minimal pericardial effusion and SARS-CoV-2 infection. She received treatment with low molecular weight heparin (1 mg/kg/dose–6000 UI/dose, 2 doses/day), followed by treatment with acenocoumarol. The initial dosage of acenocoumarol was 6 mg/day, being adjusted depending on international normalized ratio (INR) for 6 months. It ranged between 4.5–6 mg/day. INR has been monitored and maintained around the value of 2. In addition to the treatment recommended by the GP, the prescribed medication during the hospitalization consisted of vitamin B6 50 mg/day and vitamin B1 100 mg/day, as well as antibiotics, (Ceftriaxone 1 g × 2/day for 10 days), as indicated by the otolaryngologists (in addition to the aforementioned antithrombotic treatment).

The evolution was favorable, with the absence of headache. The cardiac exam repeated after 2 weeks since the discharge was normal with normal ultrasound and normal electrocardiogram. Cerebral magnetic resonance angiography was repeated after 3 and 6 months respectively. The first showed minor post thrombotic sequelae at the level of the right transverse sinus. The latter showed normal venous flow. An MRI evaluation after 12 months, since the cerebral venous thrombosis diagnosis, was not possible because the patient had turned 18 years old, therefore she could no longer be investigated in our clinic.

## 3. Discussion

Cerebral venous thrombosis in children may have various causes. The most common conditions associated with pediatric cerebral venous thrombosis and predisposing conditions are presented in Table 1 [4,11]. Apart from the aforementioned etiologic agents, in neonates there may be other factors, namely maternal causes such as chorioamnionitis and diabetes, hypertension, or perinatal causes (meconium aspiration, Apgar score < 7 at the five-minute mark, respiratory distress which requires intubation, neonatal infection, polycythemia, severe dehydration, respiratory tract infection, disseminated intravascular coagulation, congenital diaphragmatic hernia) which favor cerebral venous thrombosis to occur [4].

In the last 2 years, after the identification of the new SARS-CoV-2, cases of cerebral venous thrombosis have been reported in medical literature in adult patients with COVID-19, however there are only two studies which present cases of COVID-19 associated with cerebral venous thrombosis in patients aged less than 18 years old and one case with a boy aged 18 who was diagnosed with cerebral venous thrombosis secondary to SARS-CoV-2 infection (Table 2). The manifestation of clinical signs which raise suspicion of cerebral venous thrombosis generally occurred 3 to 14 days after the debut of the infection [14,15]. Dakay et al. (2021) reported the case of a 17-year-old teenager with obesity that tested positive for SARS-CoV-2 two weeks prior accusing headache (left hemicrania) and nausea. Neuroimaging indicated thrombosis of the left transverse venous sinus, sigmoid sinus, jugular vein, and a segment of the right transverse sinus [14]. Essajee et al. (2020) present the case of a female patient aged 2 years and 7 months with tuberculosis meningitis and SARS-CoV-2 infection that is diagnosed with extensive cerebral sinus venous thrombosis on the 8th day since the viral infection [15]. Asif et al. (2020) present the case of an 18-year-old male patient who was hospitalized for persistent global headache, the debut of which was 1 week since the full recovery after COVID-19. Initially, it was considered to be a residual symptom of the viral infection, but the patient’s condition worsened over time and CT venogram showed cerebral sinus venous thrombosis [16]. In comparison, our patient presented headache (right hemicrania) from the debut of the COVID-19 infection, however cerebral venous thrombosis was diagnosed on the 9th day, when she was hospitalized and MRI of the brain was performed. Laboratory tests excluded other etiological causes of cerebral venous thrombosis; therefore, it was considered that it represented a complication of SARS-CoV-2 infection.

The causes of neurological complications in pediatric patients infected with SARS-CoV-2 are not completely understood at the present time. Some authors agree that the mechanism by which COVID-19 may cause neurological manifestations is represented by direct infection of the nervous system [17] and its vasculature and inflammatory responses secondary to local and/or systemic infection [18,19]. Lima et al. (2020) consider that SARS-CoV-2 is a neurotropic, neuroinvasive and neurovirulent virus [20]. Dewanjee et al. (2021) suggest four pathways in which SARS-CoV-2 can reach the central nervous system (CNS): neuronal, hematogenic, lymphatic, and through infected immune cells [21].

In the neuronal pathway, the virus reaches the terminal buttons, where it replicates. It is then transported towards the soma, invading the CNS [22]. Thus SARS-CoV-2 may reach CNS predominantly by way of the olfactory tract in the first stages of the infection [23] or by way of the trigeminal nerve or the vagus nerve, which innervate different parts of both the upper and the lower respiratory tract [24]. In addition, it has been demonstrated that Coronaviruses can invade the CNS following the synapse-connected route by infecting peripheral nerve endings [20,25]. Hoffmann et al. (2020) consider that SARS-CoV-2 can determine neuronal effects by binding to the angiotensin-converting enzyme 2 receptor (ACE2), being able to be actively transported or to diffuse at the level of the axon [26].

The hematogenic pathway has been proposed because it has been discovered that epithelial alveolar type II cells and cells of the gastrointestinal tract possess ACE2 receptors and SARS-CoV-2 binds to the ACE2 receptors, produces damage of the epithelial barrier and enters the blood circulation [20,27]. Once in the circulation, the virus can reach the cerebral level by the bond between its spike protein and ACE2 receptors of either the endothelial cells of the blood-brain barrier (BBB) or of the epithelial cells of the blood-cerebrospinal fluid barrier (BCSFB) in the choroid plexuses [27]. Lima et al. (2020) are of the opinion that SARS-CoV-2 can cross BBB by inducing an inflammatory or hypoxic reaction, releasing proinflammatory cytokines [20]. Achar and Ghosh (2020) identified tumor necrosis factor-alpha (TNF-α), interferon-gamma (IFN-γ), interleukin-2 (IL-2), interleukin-6 (IL-6) and interleukin-8 (IL-8) as being the most important proinflammatory mediators involved in the cellular invasion of SARS-CoV-2 [28].

Because the lymphatic system is well-represented at the level of the trachea and bronchi, it has been proven that lymphatic endothelia present CD209L receptors. These receptors, also named L-SIGN, are another type of molecule which allow the binding of SARS-CoV-2; therefore, there are authors who propose that the virus can indirectly reach the level of CNS indirect, in the lymphatic pathway [27,29].

Numerous authors consider that SARS-CoV-2 can reach CNS by way of infected immune cells (T lymphocytes, monocytes and neutrophils) which cross BBB and can simultaneously represent true reservoirs for viral particles [30,31]. This hypothesis is to be taken into consideration because sequences of viral RNA have been present in the bronchoalveolar lavage of patients with COVID-19 infection, as stated by Bost et al. (2020) [32].

Other authors are of the opinion that the neurological damage in SARS-CoV-2 infection is secondary to cardiovascular complications and cerebral hypoxic events caused by respiratory distress, following the primary pulmonary affection [33,34].

It has been proposed that SARS-CoV-2 infection may be the cause of endothelial lesions [35,36,37], hyperinflammatory state [36,38], and platelet activation [36,39], leading to cerebral venous thrombosis. Data of the literature describes several mechanisms by which SARS-CoV-2 can determine these CNS lesions. Najjar et al. (2020) consider that SARS-CoV-2 can activate an immune response which leads to the secretion of proinflammatory mediators (TNF-α, IFN-γ, IL-2, IL-6, Il-7) and cytokines. This increases the permeability of the BBB, which eventually creates a neuroinflammatory microclimate that negatively influences neurotransmission and is favorable for the neuronal toxicity mediated by glutamate [40]. Helms et al. (2020) stated that SARS-CoV-2 affects the neurovascular endothelia directly because of the interaction between the viral surface spike-glycoprotein (S1) and ACE2 receptors of the vascular endothelial cells, which leads to vascular lesions which cause a decrease in cerebral perfusion [41]. Some authors are of the opinion that the interaction between the new Coronavirus and ACE2 receptors can favor a proinflammatory and prothrombotic status, therefore determining vasculitis lesions or damage to the vascular membrane and the activation of the coagulation cascade [42,43,44]. In addition, Saavedra (2005) considers that ACE2 receptors at the CNS level play a significant role in sympathoadrenal system regulation. This seems to be the reason why the interaction between the virus and these receptors can disturb the autoregulation of intracranial pressure [44]. Moreover, SARS-CoV-2 can repress the expression of ACE2 and increase angiotensin II at the level of the cerebrum, which can determine both neuronal and neuroendothelial dysfunction [45].

In our case, because both cardiac and pulmonary clinical manifestations were minor, the cerebral venous thrombosis associated with SARS-CoV-2 infection was most probably produced by vascular endothelial lesions which are favorable for a proinflammatory and prothrombotic status, with vasculitis lesions or damage of the integrity of the vascular membrane leading to activation of the coagulation cascade.

In patients with venous thromboembolism in association with COVID-19, laboratory tests indicate high levels of prothrombotic markers (fibrinogen and D-Dimer), and inflammatory markers (C reactive protein, IL-6), which are related to hypercoagulable state [46,47]. In the case of the female patient diagnosed with SARS-CoV-2 infection and extensive cerebral sinus thrombosis presented by Essajee et al. (2020), the C-reactive protein level was high, coagulation studies revealed an elevated international normalized ratio, elevated prothrombin time, normal activated partial thromboplastin time, elevated fibrinogen, elevated D-Dimer as well as elevated ferritin [15]. In the case of the teenager diagnosed with COVID-19 and extensive dural venous sinus thrombosis, the laboratory tests highlighted elevated D-Dimer, but the other coagulation parameters were in normal ranges [14]. For the 18-year-old adolescent diagnosed with cerebral venous thrombosis secondary to COVID-19 in the study conducted by Asif et al. (2020) investigations showed normal cell counts with normal platelets, coagulation profile was normal and elevated C reactive protein [16]. In our case, the patient showed interstitial pneumonia signs and her levels of C-reactive protein, ferritin, and D-Dimer were initially elevated.

In general, the clinical presentation of a pediatric patient with cerebral venous thrombosis is not specific, the most frequent neurological symptoms being presented in Table 3 [4,48].

Moreover, symptoms such as respiratory failure and jittery movements in neonates can be part of the clinical presentation of a patient with cerebral venous thrombosis in this age group [4]. Saposnik et al. (2011) consider that the main clinical signs of cerebral venous thrombosis can be classified into two categories. Firstly, there are symptoms caused by impaired venous drainage which leads to a rise in intracranial pressure. Secondly, there are symptoms which occur due to cerebral lesions caused by ischemic events [49]. Moreover, it has been noticed that clinical signs depend on the localization of the thrombosis. For instance, cerebral thrombosis which is limited to the level of the superior sagittal sinus—which is one of the most frequent localizations found in pediatric patients—has the following clinical manifestations headache and papilledema, which are caused by the increased intracranial pressure, in association with epileptic seizures and motor deficit [49]. In addition, in the case of lateral sinus thrombosis the clinical symptoms also appear due to an increased intracranial pressure. Another example would be thrombosis of the deep cerebral venous system, which can determine thalamic or basal ganglia infarction with akinetic mutism and rapid neurological deterioration with coma [49]. Cortical vein thrombosis is a very rare condition and it clinically manifests with epileptic seizures and motor deficit [50].

Ghosh et al. (2021) consider that the clinical presentation of cerebral venous thrombosis associated with Covid infection consists of encephalopathy symptoms, signs of increase in intracranial pressure or focal deficits which also appear in cerebral venous thrombosis with other etiologies [13]. In the case presented by Essajee et al. (2020), neurological signs were represented by depressed level of consciousness with Glasgow Coma Scale 11, pupillary asymmetry with right ptosis and left hemiplegia [15]. The neurological signs of the teenager patient monitored by Dakay et al. (2021) consisted of left hemicrania and blurred vision with no focal neurologic deficits [14]. In the case presented by Asif et al. (2020), the only neurological symptom was persistent and severe global headache with associated mild photophobia [16]. The clinical presentation of our patient included signs of intracranial hypertension, headache since the debut, followed by vomiting which the GP considered to be a sign of gastroenteritis.

Neurological manifestations in children in the context of SARS-CoV-2 infection may appear before other signs specific for the viral infection or may even be the only clinical manifestations of COVID-19. In the case presented by Dakay et al. (2021), headaches associated with vision disturbance were the sole clinical signs of SARS-CoV-2 infection [14]. In the case of the female patient presented by Esajee et al. (2020), the neurological symptoms were severe since the admission (depressed level of consciousness, with a Glasgow Coma Scale of 11, right pupillary dilatation with right ptosis, left hemiplegia), whereas the signs of pulmonary damage appeared only after 72 h since the admission—they were also severe, and the patient needed hight flow nasal oxygen and dexamethasone [15]. Similarly, our patient accused headache since the debut of the viral infection and this was the main symptom and her respiratory manifestations were minor, compared to the patient in the study conducted by Asif et al. (2020), who had presented fever, cough and myalgia since the debut of COVID-19, and the headache aggravated 1 week after [16]. Siracusa et al. (2021) propose three possible situations in which neurological complications can appear in the context of SARS-CoV-2 infection in children. In the first situation, neurological signs appear during COVID-19 [12], as in the case presented by Dakay et al. (2020), the case of the girl presented by Esajee et al. (2020), and in the case of our adolescent. The second situation consists of the occurrence of neurological signs immediately after the recovery from the viral infection [12], as objectified by Asif et al. (2020), where headache aggravated after 1 week, being initially misdiagnosed as residual symptom of his recovery from COVID-19, especially because the swab test was negative. The third situation is represented by neurological symptoms which appear in the context of multisystem inflammatory syndrome in children [12].

In cerebral venous thrombosis the most important paraclinical investigation is represented by neuroimaging techniques. The diagnosis is established by observing a lack of flow in the cerebral veins. Computed tomography scans may detect deep venous thrombosis, however this method is less accurate, the diagnosis being missed in up to 40% of cases [6,51,52]. Cerebral magnetic resonance angiography or computed tomography angiography are the exploration that defines the involved venous structures with great accuracy, highlights any possible local causes (cerebral infections, cerebral tumors or mastoiditis), evaluates associated cerebral lesions (cerebral edema) or complications (hemorrhagic transformation). Those are the reasons why Ghosh et al. (2021) insist on the importance of brain MRI in patients with Covid-19 infection and neurological manifestation [13]. In the patient of Dakay et al. (2021) MRI brain with MR venography revealed extensive dural venous sinus thrombosis involving the straight sinus, bilateral transverse sinus, sigmoid sinus and superior sagittal sinus [14]. In the case of the girl presented by Essajee et al. (2020) CT brain scan with contrast revealed multiple filling defects in the venous system, mainly superior sagittal sinus and the transverse sinuses [15]. The adolescent evaluated by Asif et al. (2020) initially performed a CT scan without contrast substances which highlighted hyperdense internal cerebral veins. This raised the suspicion of cerebral venous thrombosis, confirmed by the CT venogram that was later realized [16]. In our case, cerebral CT also raised the suspicion of cerebral thrombosis, which was confirmed by MRI angiography.

In the adult population with COVID-19 it has been demonstrated that the presence of comorbidities has an unfavorable influence for the patients’ prognosis. In a retrospective study which included 841 patients with COVID-19, Reddy et al. (2020) observed that the association of hypertension, diabetes, obesity, dyslipidemia, heart disease, tobacco smoking and disease severity represent risk factors for the occurrence of neurological complications [53]. In the aforementioned cited studies, two pediatric patients presented comorbidities (one boy with obesity [14] and one girl with tuberculosis [15]), and one boy presented no associated medical conditions [16], as did our patient. She did not present any chronic diseases or risk factors for thrombosis or associated with a severe evolution of SARS-CoV-2 infection. Prognosis of pediatric patients diagnosed with SARS-CoV-2 infection and cerebral venous thrombosis is quite variable. Dakay et al. (2021) mention favorable outcome under treatment in their adolescent patient who was discharged with antithrombotic treatment [14]. After 2 weeks he was hospitalized again for transient vision disturbance, but the repeated neuroimaging exam did not reveal any new lesions [14]. The evolution of the patient of Asif et al. (2020) was favorable; the antithrombotic treatment was maintained for 3 months. The remission of the headache was almost complete after 2 weeks since discharge [16]. Moreover, the prognosis of the female patient’s case presented by Essajee (2020) was favorable under treatment: she was discharged with residual left hemiparesis and was recommended to undergo physiotherapy and occupational therapy [15]. In our case, the patient received low molecular weight heparin followed by acenocoumarol for 6 months, with good results both clinically and radiologically.

## 4. Conclusions

Even if SARS-CoV-2 virus generally affects the respiratory tract, in some cases neurological signs such as intracranial hypertension can be the first or the most significant symptoms in pediatric patients. The reason why in this group of patients it is advised to perform brain MRI is that cerebral venous thrombosis may represent a complication of SARS-CoV-2 infection.

## Figures and Tables

**Table 1:** Conditions associated with cerebral venous in children.

Cerebral infection (meningitis, cerebral abscess)
Ear, nose and throat (ENT) infections (otitis media and mastoiditis, sinusitis)
Neurosurgical causes (central nervous system tumors, head trauma, post intracranial surgery, hydrocephalus with or without ventriculoperitoneal shunt)
Anemia (caused by iron deficiency, sickle cell disease, thalassemia, autoimmune hemolytic anemia, paroxysmal nocturnal hemoglobinuria)
Malignancy (leukemia, lymphoma)
Cardiac disease (cyanotic congenital heart disease, post-heart surgery, post catheterization)
Autoimmune disorders (Behçet disease, systemic lupus erythematous, antiphospholipid antibody syndrome, inflammatory bowel disease, thyrotoxicosis, Cushing syndrome, idiopathic thrombocytopenic purpura)
Renal disease (nephritic syndrome, hemolytic-uremic syndrome)
Metabolic conditions (diabetic ketoacidosis, homocystinuria)
Chromosomal disorders (Down syndrome)
Medication (L-Asparaginase, oral contraceptives, corticosteroids)
Predisposing conditions (dehydration, fever, hypoxic-ischemic injury, post-lumbar puncture, antithrombin III deficiency, protein C deficiency, protein S deficiency, resistance to activated protein C and factor V Leiden)

**Table 2:** Reported cases of cerebral venous thrombosis during COVID-19 infection in children [14,15,16].

Authors (Year)	Dakay et al. (2021)	Essajee et al. (2020)	Asif et al. (2020)
Age/ sex	17 y/M	2 y and 7 mo/F	18 y/M
Comorbidities	Obesity	Tuberculosis	None
Neurological symptoms	Headaches with occasional emesis, blurred vision	Depressed level of consciousness, pupillary asymmetry with right ptosis and left hemiplegia	Headache and photophobia
Respiratory or other symptoms	None	No respiratory symptoms at admission, respiratory distress gradually developing after 72 h	None—at the hospitalization for persistent global headache.
Serology SARS-CoV-2	Positive	Positive	Negative (previous diagnosis of COVID-19 with confirmed positive throat swab PCR test)
Radiology brain investigations	MRI venography: extensive dural venous sinus thrombosis	CT with contrast: venous system thrombosis, mainly superior sagittal sinus and the transverse sinuses	CT venogram: filling defects throughout the sigmoid and transverse sinuses bilaterally, extending into straight and superior sagittal sinuses
Changes in coagulation tests	Elevated D-Dimer	Elevated INR, elevated prothrombin time, elevated fibrinogen, elevated D-Dimer, elevated ferritin	Elevated C reactive protein
Treatment	Enoxaparin	Aspirin	Low-molecular-weight heparin followed by Enoxaparin
Outcome	Favorable Hospitalized again after 2 weeks for transient vision disturbance, but repeat neuroimaging revealed improving of thrombotic lesions	Favorable Recovering with physiotherapy and occupational therapy for a residual left hemiparesis	Favorable After 2 weeks almost complete resolution of headache.

F-female; M-male; mo-months; MRI-magnetic resonance imaging; CT-computerized tomography; INR-international normalized ratio.

**Table 3:** Symptoms and signs of cerebral venous thrombosis in children.

Epileptic seizures (focal or generalized)
Altered state of consciousness and coma
Focal neurological deficits (cranial nerve paralysis, hemiparesis, hemisensorial disorders)
Eyesight disorders
Visual impairment (transient obscurations, reduced acuity, blindness)
Papillary edema
Speech impairment
Headache or other signs of increase in intracranial pressure (nausea, vomiting)
Ataxia
Acute psychiatric symptoms

## Data Availability

Not necessary.
